# Outcome of protease inhibitor substitution with nevirapine in HIV-1 infected children

**DOI:** 10.1186/1471-2334-8-144

**Published:** 2008-10-22

**Authors:** M Isabel Gonzalez-Tome, Jose Tomas Ramos Amador, M Jose Mellado Peña, M Luisa Navarro Gomez, Pablo Rojo Conejo, Pablo Martin Fontelos

**Affiliations:** 1 Division of Immunodefiencies. Hospital 12 de Octubre. Madrid. Spain; 2 Paediatrics Department. Hospital de Getafe. Madrid. Spain; 3 Paediatrics Department. Hospital Carlos III. Madrid. Spain; 4 Paediatrics Department. Hospital Gregorio Marañón. Madrid. Spain

## Abstract

**Background:**

Protease inhibitors (PIs) have been associated with metabolic complications. There is a trend to switch to simpler therapy to improve these disturbances. We report a case-series describing the effects in metabolic abnormalities in seven HIV-infected children, previously treated with protease inhibitor (PI) after switching to nevirapine.

**Methods:**

Seven children with stable PI-containing regimen and a long lasting HIV-1 RNA < 50 copies/ml were switched to nevirapine. All patients were naïve to non nucleoside reverse transcriptase inhibitor. PIs were switched to nevirapine. Preentry nucleoside reverse transcriptase inhibitors were maintained. The substitution of PIs with nevirapine was made when the patient showed hyperlipidemia or lipodystrophy or the physician and/or the patient's willingness to simplify. Clinical, laboratory data and anthropometric parameters were assessed every 3 months. Dual-energy X-Ray absorptiometry scans (DXA) was performed at baseline and at 12 months.

**Results:**

Seven HIV-infected children were enrolled. Median age: 130 months (99,177). Median baseline CD4%: 32%. All had HIV-1 RNA < 50 copies/ml. Median length of preentry PI-therapy was 47 months (28, 91). Median age at the beginning of nevirapine was 120 months (99,177). Median decrease in cholesterol in 7.2 mmol/L was observed (P = 0.09), from baseline to 12 months. HDL-cholesterol increased in 5.1 mmol/L (P = 0.03) throughout the study period. No significant changes were observed in DXA with regard to body fat, but changes in total body bone mineral content and lean body content were significant. CD4% remained stable. All patients but one maintained viral load < 50 copies/ml at 12 months. The patient with virologic failure referred bad adherence. Children referred to take medication more easily.

**Conclusion:**

PI substitution with nevirapine improved lipid profile in our patients, although this strategy did not show significant changes in body fat or lipodystrophy.

## Background

Metabolic complications are prevalent in HIV-infected children treated with highly active antiretroviral therapy (HAART). Nowadays, lipodystrophy and osteopenia had also been reported in HIV-infected children [[1-7], and [8]]. Lipodystrophy syndrome is characterized by physical and metabolic abnormalities including fat redistribution, dyslipidemia and insulin resistance. Protease inhibitors (PIs) have been associated with the development of these events. This is one of the most important limiting factors for long term therapy with HAART [[9-11], and [12]]. There is a trend to switch to simpler therapy including efavirenz, nevirapine, abacavir or atazanavir-containing regimen that might maintain viral suppression and improve metabolic disturbances [13,14]. In adults, there are reports demonstrating that the replacement of PI by nevirapine might improve lipid abnormalities [[15-18], and [19]]. There are few studies documenting these strategies in children. In our case-series, we describe the possible effects in metabolic abnormalities, including lipid profile, lipodystrophy (LD) and bone mineral density (BMD) in seven HIV-infected children, previously treated with PI after switching to nevirapine and with a high level of viral suppression for a long period of time (at least 6 months).

## Methods

Children's regimens were changed if they fulfilled these criteria: all were older than 12 months; they were receiving a stable PI-containing regimen (2 nucleoside reverse transcriptase inhibitor (NRTI) plus 1 or 2 PIs) for at least 6 consecutive months; they were naive to non nucleoside reverse transcriptase inhibitor (NNRTI). All patients had plasma HIV-1 RNA viral load < 50 copies/ml at baseline and for a median of 6 months before study entry and a stable clinical and immunological situation. Then, PIs were switched to nevirapine and preentry NRTI were maintained. The substitution of PIs with nevirapine was made if the patient showed hyperlipidemia and/or lipodystrophy or if the physician or the family wanted to simplify the antiretroviral regimen. Hyperlipidemia was considered if cholesterol was ≥ 6.5 mmol/L and/or LDL-cholesterol was ≥ 4 mmol/L and/or triglycerides were ≥ 5.5 mmol/L. Glucose metabolism was evaluated and was considered abnormal if fasting glucose was between 100 mg/dl and 126 mg/dl. Insulin levels and C-peptide were also performed and were considered abnormal if insulin was > 15 UI/ml and C-peptide was > 2 ng/ml. Lipodystrophy was diagnosed if patients presented at least one of the following features: peripheral loss of adipose tissue: sunken cheeks, thinning extremities, hips or buttocks (lipoatrophy), central gain in adiposity: dorsocervical and/or abdomen fat accumulation (lipohypertrophy) or both features (mixed syndrome).

Children were excluded if they suffered from hepatitis C virus infection or if they showed aminotransferases elevations or if they were receiving lipid-lowering drugs. Standard clinical and laboratory data were performed every 3 months. Laboratory data included: CD4 cell count, plasma HIV-1 RNA viral load (using Amplicor HIV monitor Roche, range 50–500.000 copies/ml), blood counts and blood chemistries (glucose, AST, calcium, phosphorus, amylase, lipase, creatinine kinase, etc). All data were obtained after 8–12 hours of fasting. No dietary or exercise history was recorded.

Metabolic evaluation and anthropometric measurements were performed every 6 months including C-peptide, insulin, lipid profile, body weight, height, mid-arm circumference, triceps and subescapular skin folds. Skin folds were measured using a manual trackball. Body weight was measured on a balance beam scale, and height was measured using the same wall-mounted stadiometer. Measurements were performed by the same person. Dual-energy X-Ray absorptiometry scans (DXA) was used to estimate whole-body composition. The same scanner (Norland XR-26HS densitometer) was used for evaluations on any patient. The entire body was scanned. Variables analysed including: bone mineral density (BMD), expressed in g/cm2, lean body mass (LBM) expressed in grams and body fat expressed in grams and as a percentage of total body weight. All compartments were analysed in the arms, trunk, legs and face. Z score for lumbar BMD and BMC-age related changes were estimated based on Zachetta's report [20]. In this study, parameters were generated from data of 778 Argentine Caucasian children (345 boys and 443 girls between 2 to 20 years old). Osteopenia was defined as a Z score less than -1 for lumbar spine, and osteoporosis when it was less than -2.5, following WHO criteria [21]. Puberty stage of patients was defined according to Tanner criteria [22]. Adherence, difficulty in taking medication and quality of life were assessed by patient and/or caregiver's self report.

The statistical software Staview Mc Intosh was used. Quantitative data were expressed in terms of means and range. Qualitative variables were expressed as percentages. Wilcoxon rank sum test for continuous data was used to determinate changes over time and statistical significance. P < 0.05 were considered statistically significant.

Written consent was obtained from six of the patients.

## Results

Seven patients were enrolled (2 girls and 5 boys), median age was 120 months (99,177). All were Caucasians. Six children acquired infection by vertical transmission. One girl had HIV infection of unknown origin. At baseline, median CD4 percentage was 33%(29–42) and all had viral load under 50 copies/ml at baseline and at least 6 months before. Related to CDC category, 2 patients were on A1, 2 on C3, 2 on B2 and one on A3. At the beginning of the follow up, 4 children were prepuberal. A girl reached stage IV at 12 years. Other 2 patients reached Tanner stage II. (Table 1) Five subjects who were born before preHAART-era had been pre-treated with NRTI as monotherapy or dualtherapy and when PIs were available were changed to HAART and 2 patients had received a PI-containing regimen as a first line therapy. All were naive to NNRTI. Median age at the beginning of first ARV therapy was: 53 months (6,119) and median age at the beginning of PIs was: 83 months (54,123). PIs used at study entry were: indinavir (3 cases), amprenavir (2 cases), ritonavir (2 cases, one was on ritonavir plus amprenavir and another was on ritonavir alone), only one was on nelfinavir. The cumulative duration of previous PI before nevirapine was: 44 months (28, 60) and the cumulative duration of mono/dualteraphy with NRTI previous PI was 52 months (0,109). (Table 2)

**Table 1:** Baseline characteristics

PATIENT	TRANSMISSION	GENDER	Age(years+months)	CATEGORY(CDC)	TANNER *	LIPODYSTROPHY
1	UNKNOWN	GIRL	13 Y 7 M	A1	III to V	no
2	VERTICAL	BOY	9 Y 10 M	A3	I-I	no
3	VERTICAL	BOY	8 Y 3 M	A1	I-I	no
4	VERTICAL	GIRL	9 Y 2 M	C3	I-I	no
5	VERTICAL	BOY	10 Y	C3	I-I	no
6	VERTICAL	BOY	14 Y 7 M	B2	II-III	lipoatrohy
7	VERTICAL	BOY	8 Y 2 M	B2	II-III	lipohypertrophy
MEDIAN			133(99,177)			

* Puberty stage. Tanner JM. Grow and adolescence (2^a^edition) Oxford. Blackwell Scientific publication. 1962

**Table 2:** Antiretroviral therapy prior to nevirapine-containing regimen

PATIENT	AGE at 1st regimen	Mono-therapy	Dual-therapy	Cumulative duration	HAART	Cumulative duration	CD4%	Viral load cop/ml
	months	NRTI	NRTI	NRTI	PI	PI	0–12 months	0–12 months
					D4T-3TC-	AMP(36 m)+		
1	119	NO	ZDV-DDI	27	AMP+RIT	RIT (6 m)	33–41	50–50
2	39	NO	ZDV-DDI	36	D4T-3TC-AMP	AMP(48 m)	32–30	50–50
3	65	NO	NO	0	ZDV-DDI-RIT	RIT(32 m)	29–34	50–50
4	14	ZDV	ZDV-DDI	19+21	D4T-3TC-NFV	NFV(56 m)	42–44	50–50
5	92	NO	NO	0	ZDV-ABC-IND	IND(28 m)	34–30	50–50
6	9	ZDV/DDI	NO	109	ZDV-DDI-IND	IND(60 m)	32–27	50–10325
7	6	ZDV	NO	92	ZDV-3TC-IND	IND(52 m)	31–34	50–50
							33%(29,42)	
median	53 m (6,119)			52 m (0,109)	130 m (99,177)	47 m (28,60)	37%(27,44)	50

NRTI: nucleoside reverse transcriptase inhibitor, ZDV: zidovudine, DDI: dianosine, D4T: stavudine, 3TC: lamivudine; PI: protease inhibitor, AMP: amprenavir, RIT: ritonavir, NFV: nefinavir, IND: indinavir; m: months; cop/ml: copies/ml (HIV-RNA, liner range: 50–500.000 cop/ml)

The substitution of PIs with nevirapine was made due to hyperlipidemia in 3 cases, the physician or the family wanted to simplify the patient' regimen in 3 cases and lipodystrophy in 1 case.

No acquired immunodeficiency syndrome-defining events were reported. No rash or aminotransferases changes occurred during this period. Only 1 patient referred mild headache at the beginning of nevirapine-containing regimen but it disappeared without discontinuing therapy. She had a prior diagnosis of migraine. Six of seven patients maintained virologic control successfully, with less than 50 copies/ml at all time points. One child showed an increase in viral load up to10.325 copies/ml at 12 months. He referred bad adherence. Median CD4% remained stable in all patients, from 33% (29, 42) at baseline to 37% (27, 44) at 12 months, (P = 0.7).

At the beginning, cholesterol was ≥ 6.5 mmol/l in 3 patients, LDL-cholesterol was ≥ 4 mmol/l in 3/6 patients and HDL-cholesterol was ≤ 1.16 mmol/l in 4/6 patients. At study entry, in all patients, triglyceride levels, fasting glucose, insulin, C-peptide, amylase or lipase were normal and did not change throughout the study period (Table 3). Median decrease in total cholesterol from baseline to 12 months was 0.7 mmol/ml (P = 0.09) and median increase in HDL-cholesterol was 0.5 mmol/ml from baseline to the end of the study (P = 0.03). No significant changes in LDL-cholesterol was observed. DXA was performed in six patients, at the study entry and in all patients at the study end. In these 6 patients, TBMC and LMB tended to increase at 12 months. About body fat changes, one girl and a boy showed a decrease in body fat and fat percentage. In another boy, there was a decrease in body fat percentage but no in total body fat. These 3 patients had also osteopenia and they were older than the rest (median age: 13 years). Therefore, TBMC and LBM, showed a significant increase (P = 0.04 and P = 0.02), but no significant changes in fat (grams or %), Z score or BMC-age related changes were founded. At baseline, one patient showed osteoporosis (Z score: -4.21) and 2 osteopenia (Z scores: -1.05 and -1.14). In 3 cases Z score were > 1 (Table 4).

**Table 3:** Metabolic parameters and lipid profile at baseline and at 12 months

PATIENT	CHOLESTEROL	HDL-cho	LDL-cho	TG	GLUCOSE	INSULIN	C-PEPTIDE
	(mg/dl)	(mg/dl)	(mg/dl)	(mg/dl)	(mg/dl)	(IU/ml)	(ng/ml)
	Values 0–12 m	0–12 m	0–12 m	0–12 m	0–12 m	0–12 m	0–12 m
1	250–182	40–47	179–127	155–36	99–97	8,9–8,4	1,1–1,6
2	192–200	NA – 65	NA – 126	70–41	91–84	4,8–5,2	0,8–1,93
3	338–245	63–69	112–160	112–59	86–94	4,6-NA	0,74-NA
4	325–290	56–61	250–193	92–177	85–86	9,6–8,9	1,8-NA
5	188–152	32–48	133–93	116–92	88–88	9,8–19,4	0,5–0,4
6	120–101	37–35	71–60	60–29	75–78	10–12,3	2,2–1,3
7	195–220	45–52	126–150	118–86	81–85	10–6,9	1,1–1,4
median at baseline	195(120,338)	45(32,63)	129(71,250)	74(60,92)	86(75,99)	9,6(4,6–10)	1,3(0,5–2,5)
median at 12 m	200(101,290)	52(35,69)	127(60,193)	74(29,177)	87(78,97)	8,6(5,2–19,9)	1,4(0,4–1,93
P	0,09	0,04	0,34	0,17	0,4	0,9	0,9

Evolution after switching.

HDL-cho: HDL-cholesterol, LDL-cho: LDL-cholesterol, TG: triglycerides

To convert cholesterol from mg/dl to mmol/l, multiply by 0.0259

To convert triglycerides from mg/dl to mmol/l, multiply by 0.0113

To convert insulin from IU/ml to pmol/l, multiply by 7.175

To convert C-peptide from ng/ml to nmol/l, multiply by 0.33

To convert glucose from mg/dl to mmol/l, multiply by 0.0555

**Table 4:** DXA at baseline and at 12 months

PATIENT	BMC(g/cm2) 0–12 m	LMB(g) 0–12 m	FAT(g) 0–12 m	FAT (%) 0–12 m	Z-SCORE 0–12 m	BMC-age related changes(%) 0–12 m
1	0,798–0.949	26.778–29.9807	13.082–12.249	31–28	-1.05 → -3.34	90.9–83.4
2	0,731–0,748	17.687–19.909	8071–10.922	30–34	2,08 → 4,31	116,9–135
3	0,759–0,832	20.751–23.293	9579–11.409	30–31	1,8 → 2,59	116,6–121,9
4	0,803–0,868	26.856–41.913	7014–5526	20-11	-4,21 → -3,01	70,1–82,1
5	0,687–0,723	19.300–20.563	7181–7949	26–27	1,01 → 0,88	110,5–110,4
6	0,76–0,83	22.559–28.061	10.967–11.393	31-28	-1,14 → -1,25	90–89,5
7	NA-0,732	NA-21.263	NA-13.069	NA-36	NA → 1,56	NA-112,8
P	0,04	0,02	0,2	0,8	0,2	0,2

BMC: bone mineral content, LMB: lean body mass, NA: not available, g: grams.

With regard to anthropometric measurements, only weight, height and mid-arm circumference showed a significant increase (P = 0.01, P = 0.02, P = 0.02), (data not shown). Lipodystrophy was clinically detected at the beginning in 2 patients, one subject showed clinical signs of peripheral lipoatrophy and another patient had central lipohypertrophy. At 12 months, there were no changes in patients with lipoatrophy, but there was a decrease in abdominal fat accumulation in the patient with central hypertrophy, this child were receiving indinavir before changing.

Patients were asked about compliance and difficulty in taking medication. All of them referred a sense of less difficulty in taking nevirapine compared with the PI-regimen.

### Summary of patients

#### Patient 1

A 13-year-old girl who was receiving d4T + 3TC + amprenavir boosted with ritonavir was simplified to d4T + 3TC + nevirapine because she suffered from hypercholesterolemia. A decrease in total cholesterol, LDL cholesterol and triglycerides was observed. She remained with VL < 50 copies/ml but her lumbar BMD Z score showed a decrease from -1.05 to -3.34.

#### Patient 2

A 9-year-old girl who was receiving d4T + 3TC + amprenavir was simplified to d4T + 3TC + nevirapine because she wanted to do her regimen easier to take. She referred that she was happier after this change. She remained with undetectable viral load. She did not show osteopenia along the follow up.

#### Patient 3

An 8-year-old-boy who was receiving ZDV + ddI + ritonavir was simplified to ZDV + ddI + nevirapine because he suffered from hypercholesterolemia. A light decrease in total cholesterol was observed but not in LDL-cholesterol. He remained with undetectable viral load and without osteopenia.

#### Patient 4

A 9-year-old girl who was receiving d4T + 3TC + nelfinavir was simplified to d4T + 3TC + nevirapine because she suffered from hypercholesterolemia. A decrease in total cholesterol and in LDL-cholesterol was observed. She showed undetectable viral load at 12 months and the osteopenia remained.

#### Patient 5

A 10-year-old boy who was receiving d4T + abacavir + indinavir was simplified to d4T + abacavir + nevirapine because he wanted to do his regimen easier to take and to avoid renal damage associated to indinavir.

#### Patient 6

A 14 year-old adolescent who was receiving ZDV + ddI + indinavir was simplified to ZDV + ddI + nevirapine because he wanted to do his regimen easier to take and to avoid renal damage associated to indinavir. At 12 months he showed a decrease in total cholesterol and LDL-cholesterol but had detectable viral load because of bad adherence. He suffered from facial lipoatrophy and it remained without changes after the substitution (we have to take into account that NRTIs were not removed and these drugs are implied in fat distribution).

#### Patient 7

An 8-year-old boy who was receiving ZDV + 3TC + indinavir was simplified to ZDV + 3TC + nevirapine because he wanted to do his regimen easier to take, to avoid renal damage associated to indinavir and to improve his abdominal lipohypertrophy. He showed an important decrease in abdominal fat accumulation at the end of the year. His viral load remained undetectable.

## Discussion

We report seven cases in which the substitution of PIs (indinavir, saquinavir and nelfinavir) with nevirapine resulted in a mild improve in total cholesterol and HDL-cholesterol. Virological maintenance was achieved in all patients but one during the 12 months. The patients that showed virological failure at 12 month referred bad adherence although the child's adherence at baseline was considered good and the new regimen was less complex. Nevertheless, all children maintained CD4 cell count and good clinical and growth parameters.

PIs may have a strong association with dyslipidemia [[2,3,5,9-12], and [23]]. There have been few studies in children to evaluate the substitution of PI with NNRTI. Mc Comsey et al reported a simplification trial in which the PI was switched to efavirenz in children who had previously undetectable viral load for more than 6 months. They showed maintenance of virologic suppression in all patients who received the new regimen. A significant improvement in lipid profile was also observed including fasting total cholesterol, LDL-cholesterol, triglycerides and total cholesterol: HDL-cholesterol ratio. The shift to a simplified 3-NRTI regimen, including abacavir, in children previously treated with PI-based HAART was reported by Castelli-Gattinara et al. In this study the new regimen allowed to maintain an immunological and virological control after 108 weeks with significant improvement of dyslipidemia, including total-cholesterol, LDL-cholesterol and triglycerides [24]. In our cases, we have only seen a significant improvement in HDL-cholesterol and a decrease in total cholesterol although this change has not been significant. We must take into account that 3 patients had been also receiving stavudine, which might be involved in dyslipidemia [25]. So, an improvement in lipid profiles have been reported when stavudine was replaced with abacavir or zidovudine in some studies. In addition, substitution of stavudine with ZDV or a non-thymidine analogue might lead to an improvement in body fat as several studies have reported [[26,27], and [28]]. Therefore, the substitution of stavudine in these children could lead to reach a better control of lipid metabolism. Additional factors that could have played a roll include diet and exercise. A low fat diet and aerobic exercise were advised at each visit, but they were not recorded [29].

Some PIs including indinavir, have been associated with insulin resistance and an improvement in insulin sensitivity has been observed in some studies when PIs were switch to NNRTI or abacavir [13,30]. In our series, none of the patients had insulin or glucose abnormalities at baseline and there were not changes in these parameters throughout the study period.

With regard to anthropometric measurements, there was an increase in weight, height and in all the skin folds from baseline to 12 months in all patients. Nevertheless, only the increases in weight, height and mild-arm circumference were significant. These changes could have been due to normal growth in these children, one of the parameter that we are not able to evaluate due to lack of a control arm.

About DXA follow up, significant changes in TBMC, LBM have been observed, but no in fat content. Therefore, in all patients there was an increase in TBMC and LMB and there was a decrease in total fat in 3 children, but changes were not significative and all gained weight with regard to baseline. Lumbar BMD Z score and BMC – age related changes did not change significantly during the study period.

Physical examinations did not show a clinical improvement in lipoatrophy. This was expected due to the continuous use of zidovudine plus didanosine in this boy. In one patient a decrease in central obesity was observed. He had been receiving Indinavir before nevirapine-regimen and this PI had been associated to central lipohypertropy [31]. These changes may be related to growth or puberty (an increase or decrease in fat or an increase in BMC and LBM should be expected during growth) or to antiretroviral itself. A control group of children maintained with the same ARV regimen including PI would be necessary to determinate the individual impact of nevirapine substitution on these parameters.

Although adherence questionnaires were not performed, our patients referred a feeling of an easier to take medication and all their new regimens contained fewer pills than the previous therapy.

This case-series includes only seven patients and 12 months may not be sufficient for a correct follow up to detect significant changes in lipid and mainly in anthropometrics parameters, specially in children and adolescents, who are experimenting continuous changes in body composition and therefore, the observed changes in these cases could be related to others factors like growth, puberty and normal physical development. Therefore, this strategy could be an option in selected children but more studies are necessary.

## Conclusion

The substitution of PI with nevirapine may be an option in selected HIV-infected children that might lead to improvement in some lipid abnormalities as other studies have showed [3,13-17].

## Abbreviations

HAART: Highly active antiretroviral therapy; PIs: Protease inhibitors; LD: Lipodystrophy; BMD: Bone mineral density; NRTI: Nucleoside reverse transcriptase inhibitor; NNRTI: Non nucleoside reverse transcriptase inhibitor; DXA: Dual-energy X-Ray absorptiometry scans; TBMC: Total body bone mineral content; LBM: Lean body mass.

## Competing interests

This study has been performed with the support of Boehringer Ingelheim Pharmaceuticals. Boehringer Ingelheim Pharmaceuticals is the manufacturer of Nevirapine.

## Authors' contributions

All the authors contributed to design the study, included patients and performed the follow up. JTRA was the principal investigator, MLNG, MJMP, PMF and PRC, also participated in the design of the study and in the follow up of the patients. All of them also contributed to write the article and have approved the final manuscript.

## Pre-publication history

The pre-publication history for this paper can be accessed here:


